# Supplementary figures and images for: Validation of CDC45 as a novel biomarker for diagnosis and prognosis of gastric cancer
Source: PeerJ. 2024 Mar 18;12:e17130. doi: 10.7717/peerj.17130 (PMC10956518; doi:10.7717/peerj.17130)

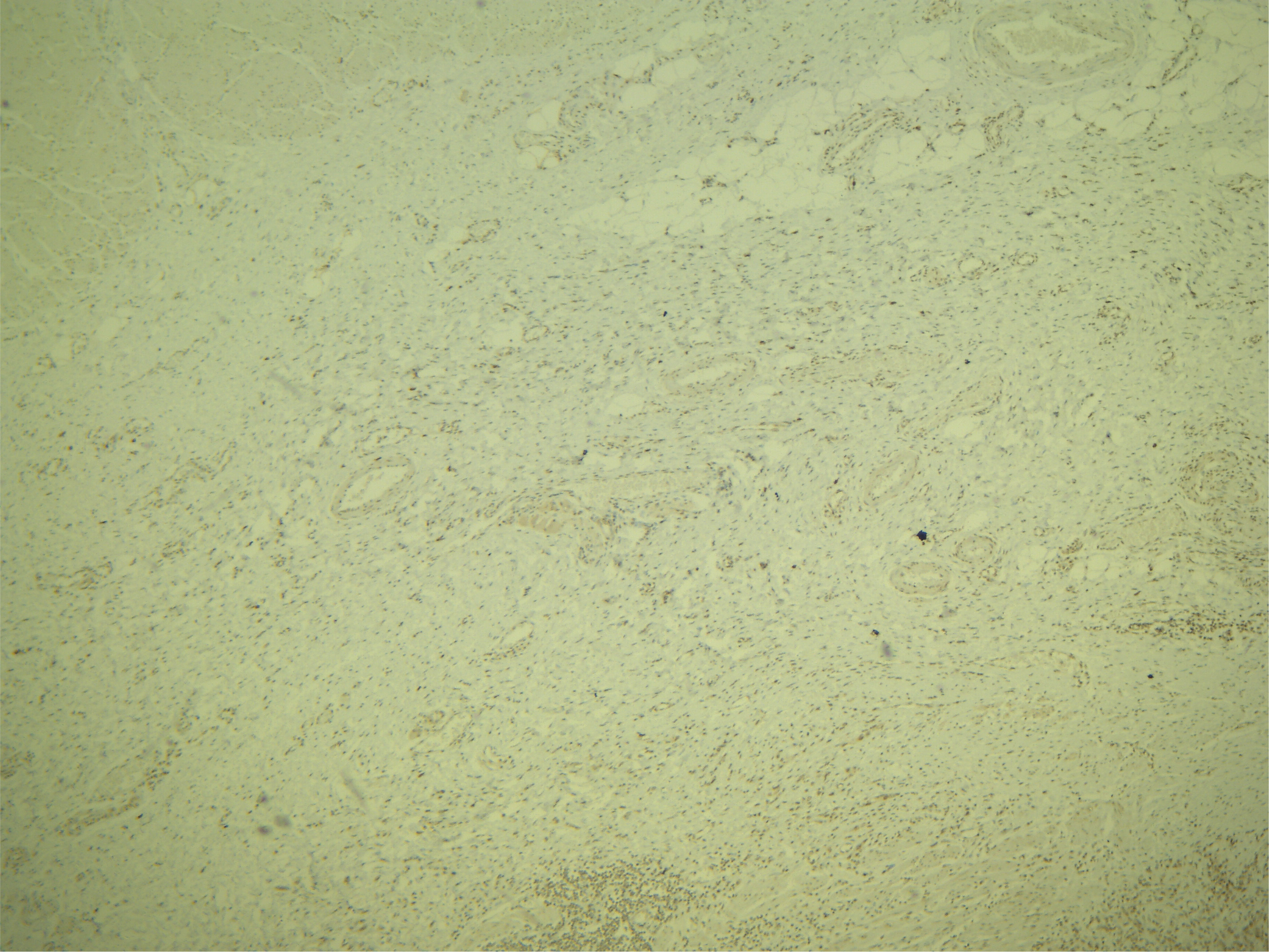

Supplement: Supplemental Information 1 [file peerj-12-17130-s001.zip › IHC-RAW-DATA/N1.jpg]

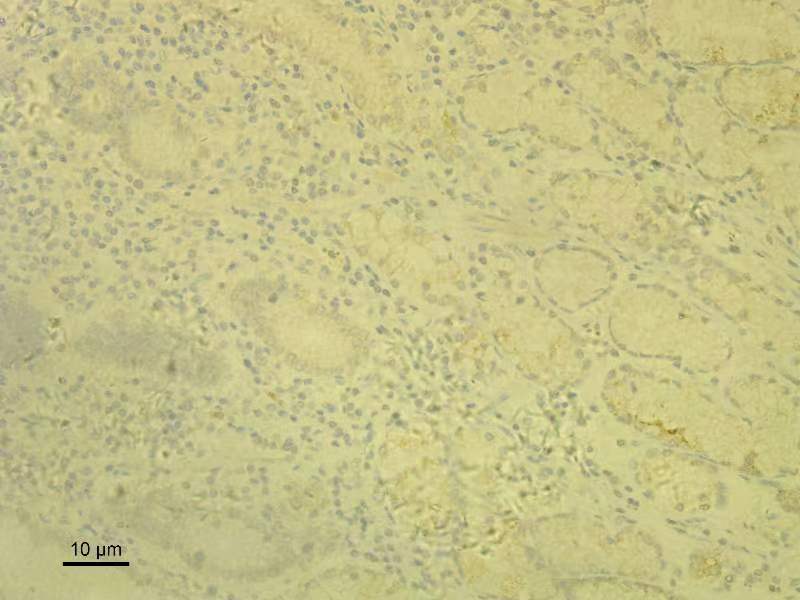

Supplement: Supplemental Information 1 [file peerj-12-17130-s001.zip › IHC-RAW-DATA/N2.jpg]

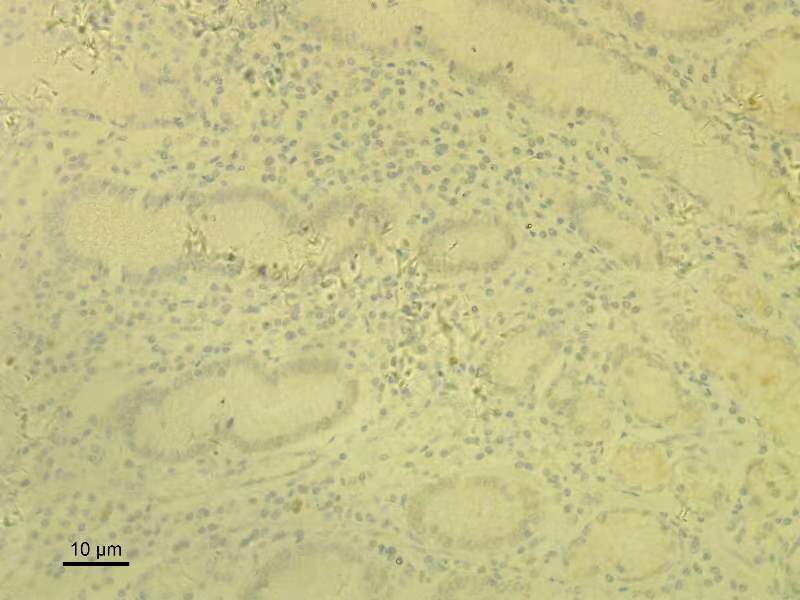

Supplement: Supplemental Information 1 [file peerj-12-17130-s001.zip › IHC-RAW-DATA/N3.jpg]

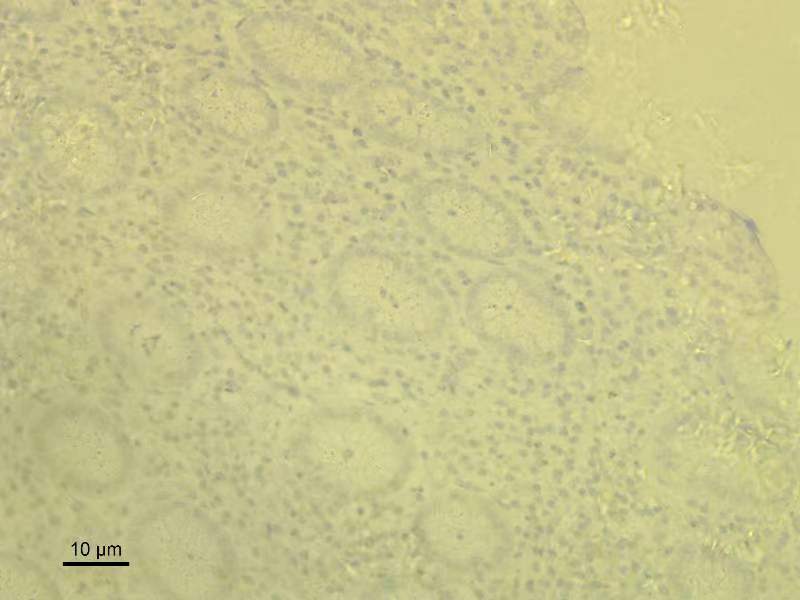

Supplement: Supplemental Information 1 [file peerj-12-17130-s001.zip › IHC-RAW-DATA/N4.jpg]

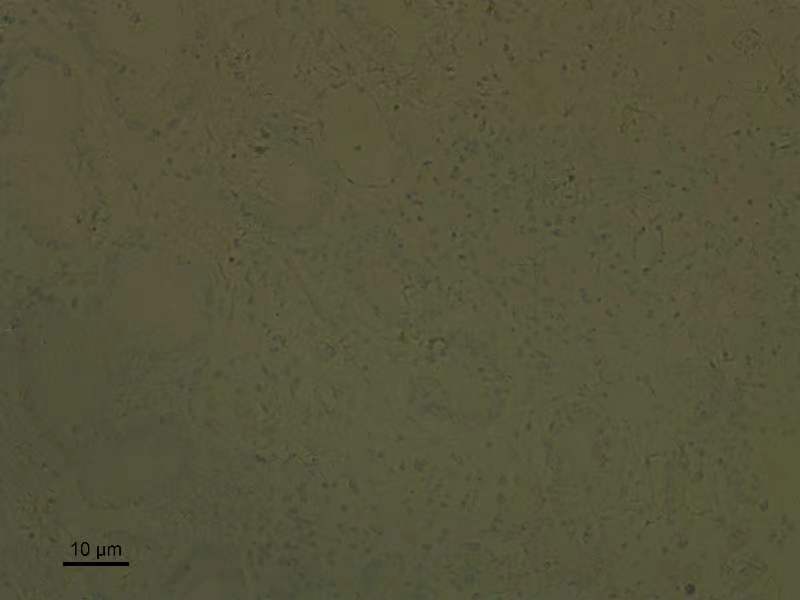

Supplement: Supplemental Information 1 [file peerj-12-17130-s001.zip › IHC-RAW-DATA/N5.jpg]

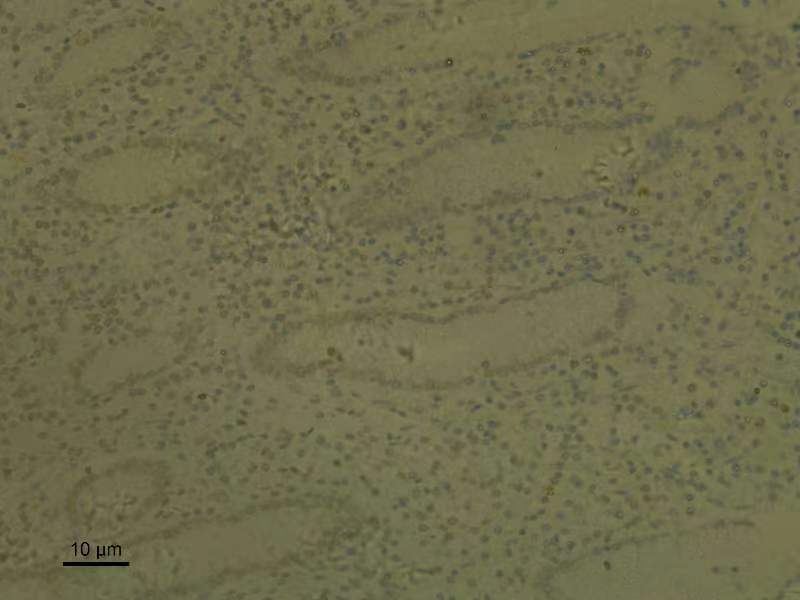

Supplement: Supplemental Information 1 [file peerj-12-17130-s001.zip › IHC-RAW-DATA/N6.jpg]

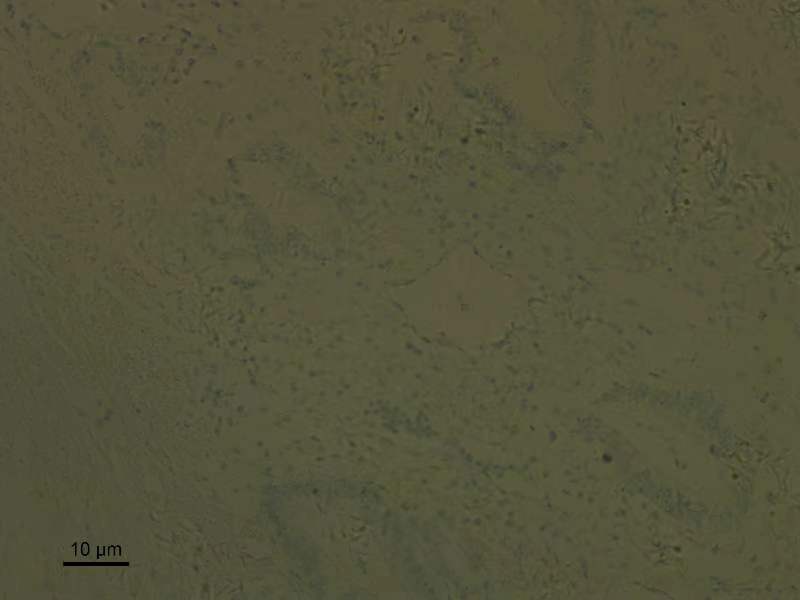

Supplement: Supplemental Information 1 [file peerj-12-17130-s001.zip › IHC-RAW-DATA/N7.jpg]

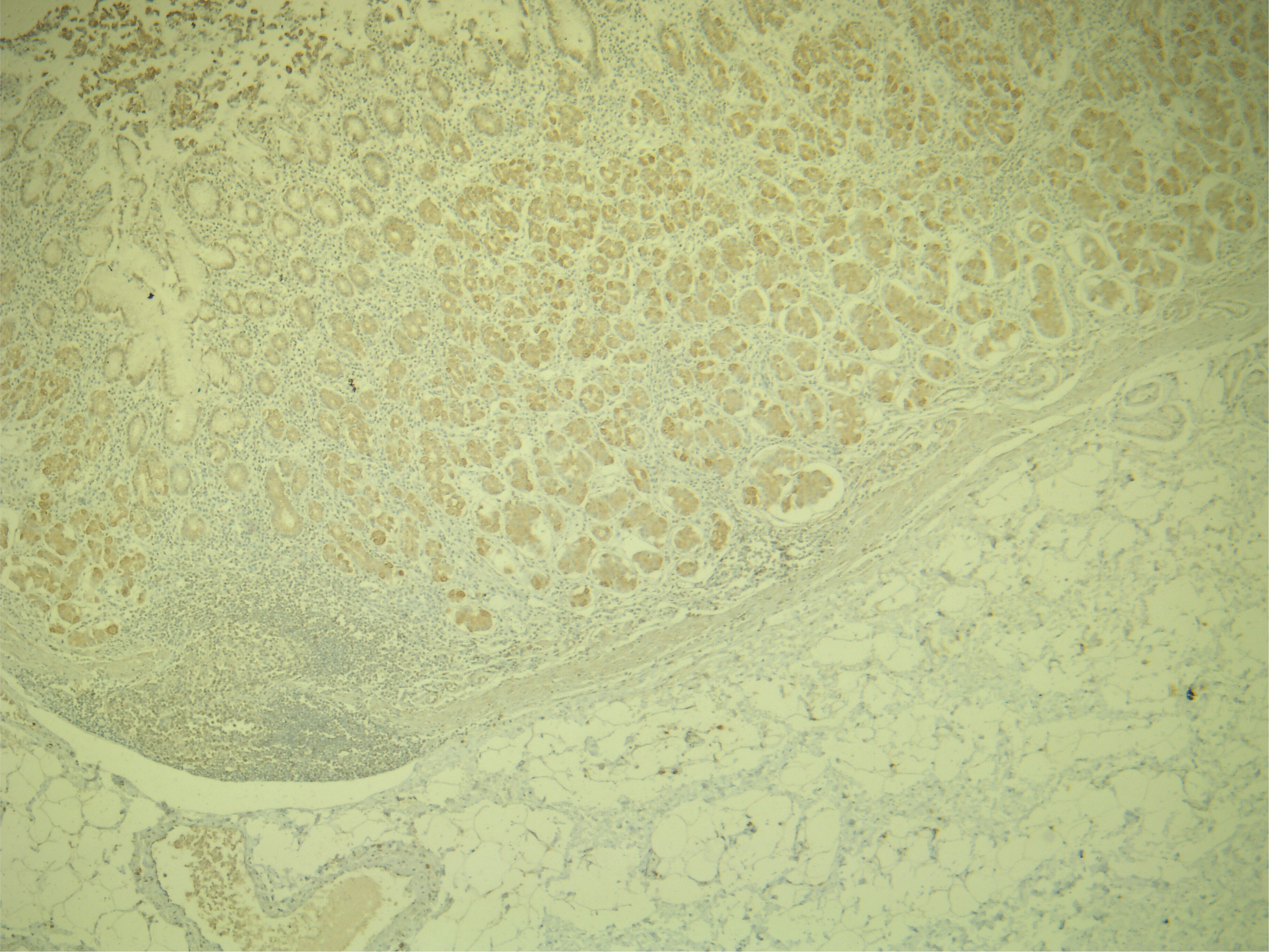

Supplement: Supplemental Information 1 [file peerj-12-17130-s001.zip › IHC-RAW-DATA/T1.jpg]

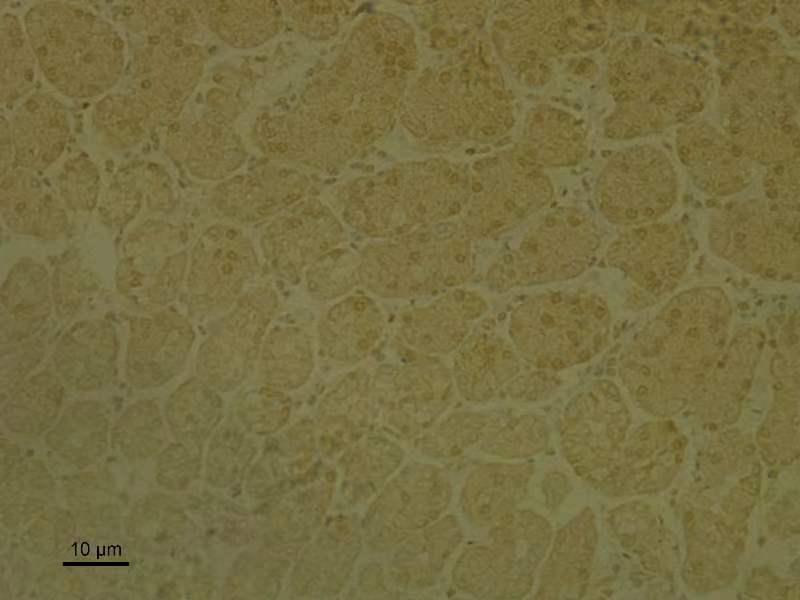

Supplement: Supplemental Information 1 [file peerj-12-17130-s001.zip › IHC-RAW-DATA/T2.jpg]

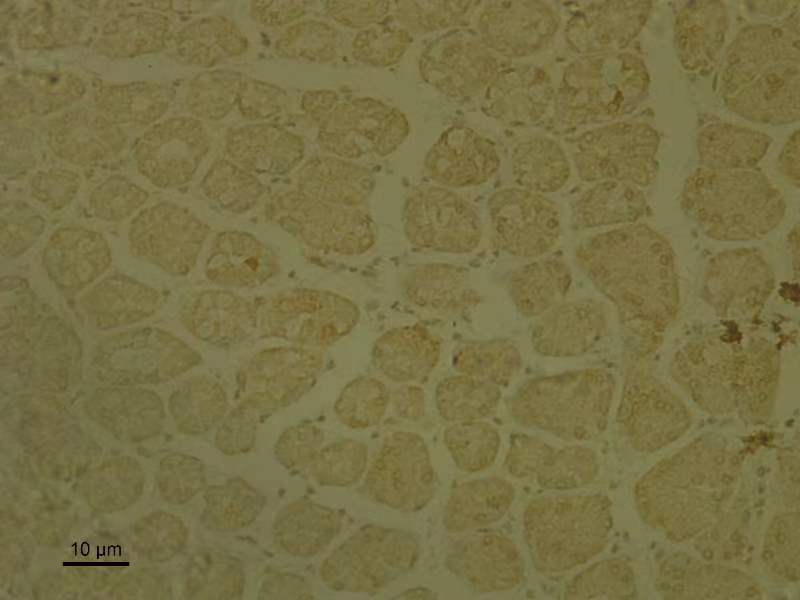

Supplement: Supplemental Information 1 [file peerj-12-17130-s001.zip › IHC-RAW-DATA/T3.jpg]

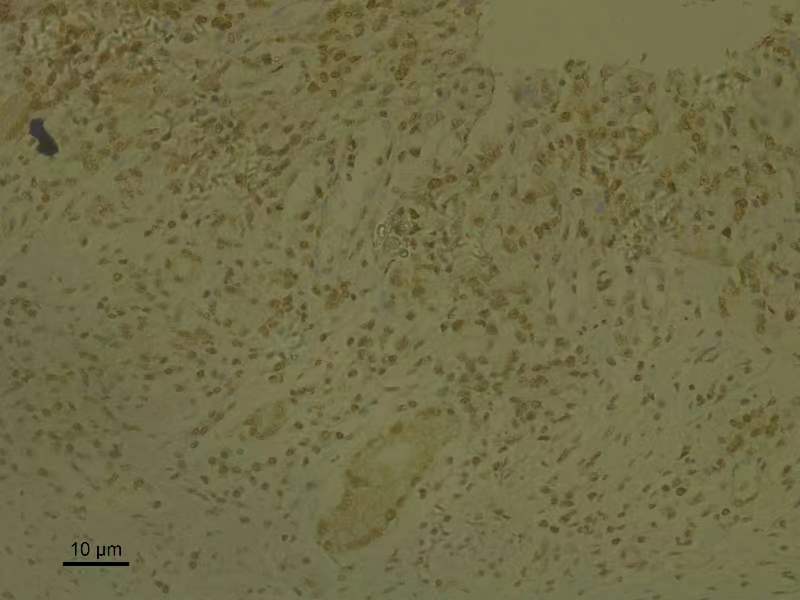

Supplement: Supplemental Information 1 [file peerj-12-17130-s001.zip › IHC-RAW-DATA/T4.jpg]

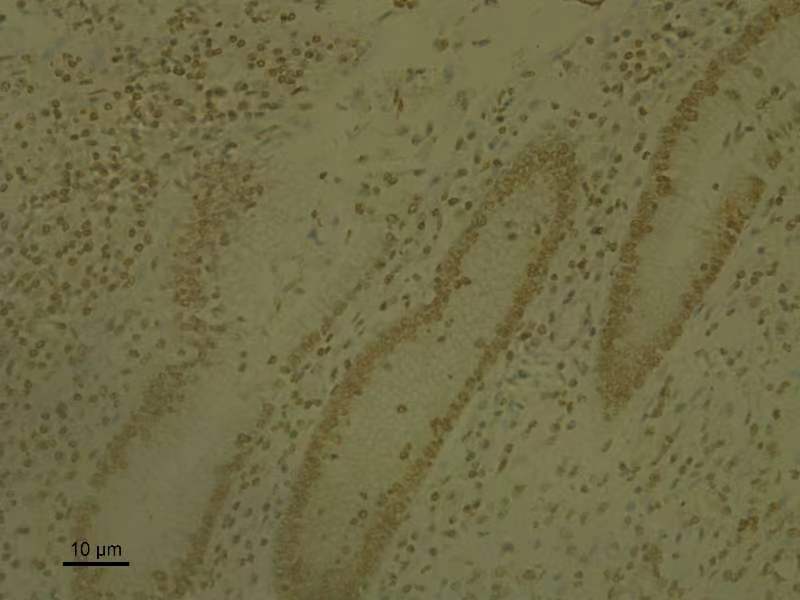

Supplement: Supplemental Information 1 [file peerj-12-17130-s001.zip › IHC-RAW-DATA/T5.jpg]

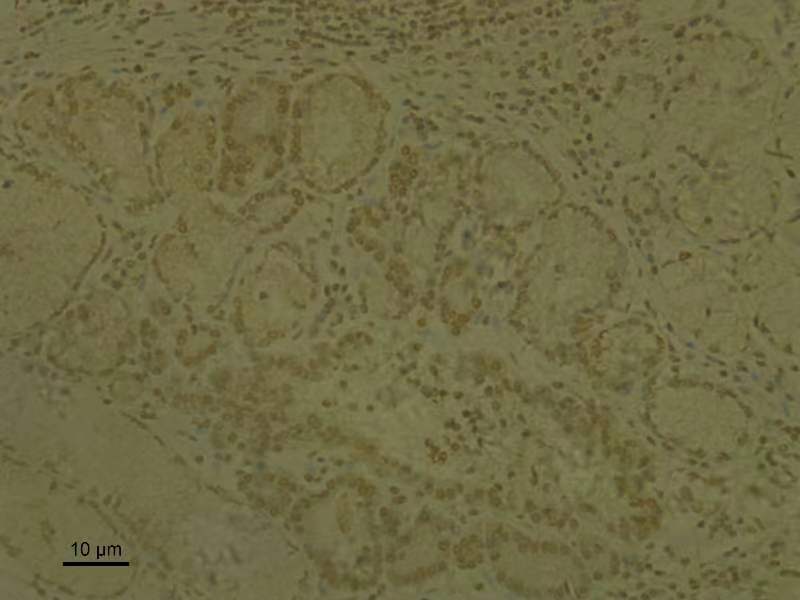

Supplement: Supplemental Information 1 [file peerj-12-17130-s001.zip › IHC-RAW-DATA/T6.jpg]

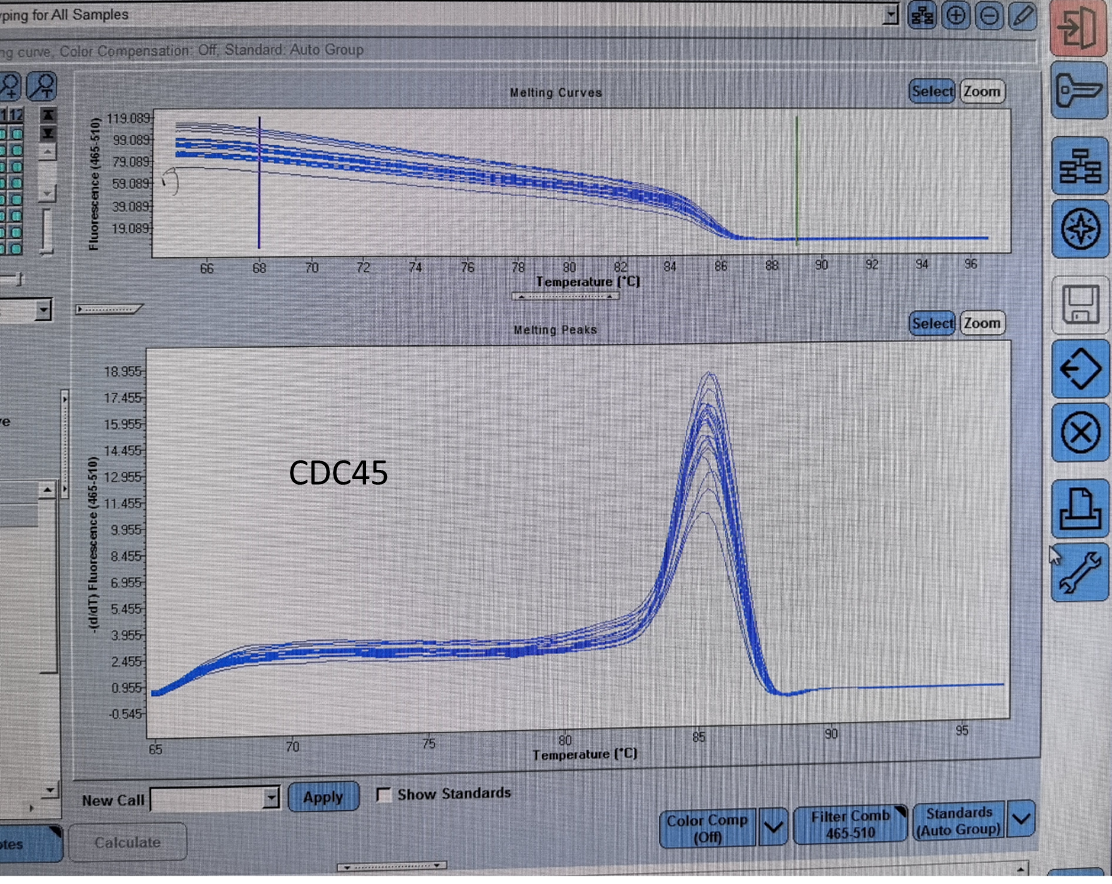


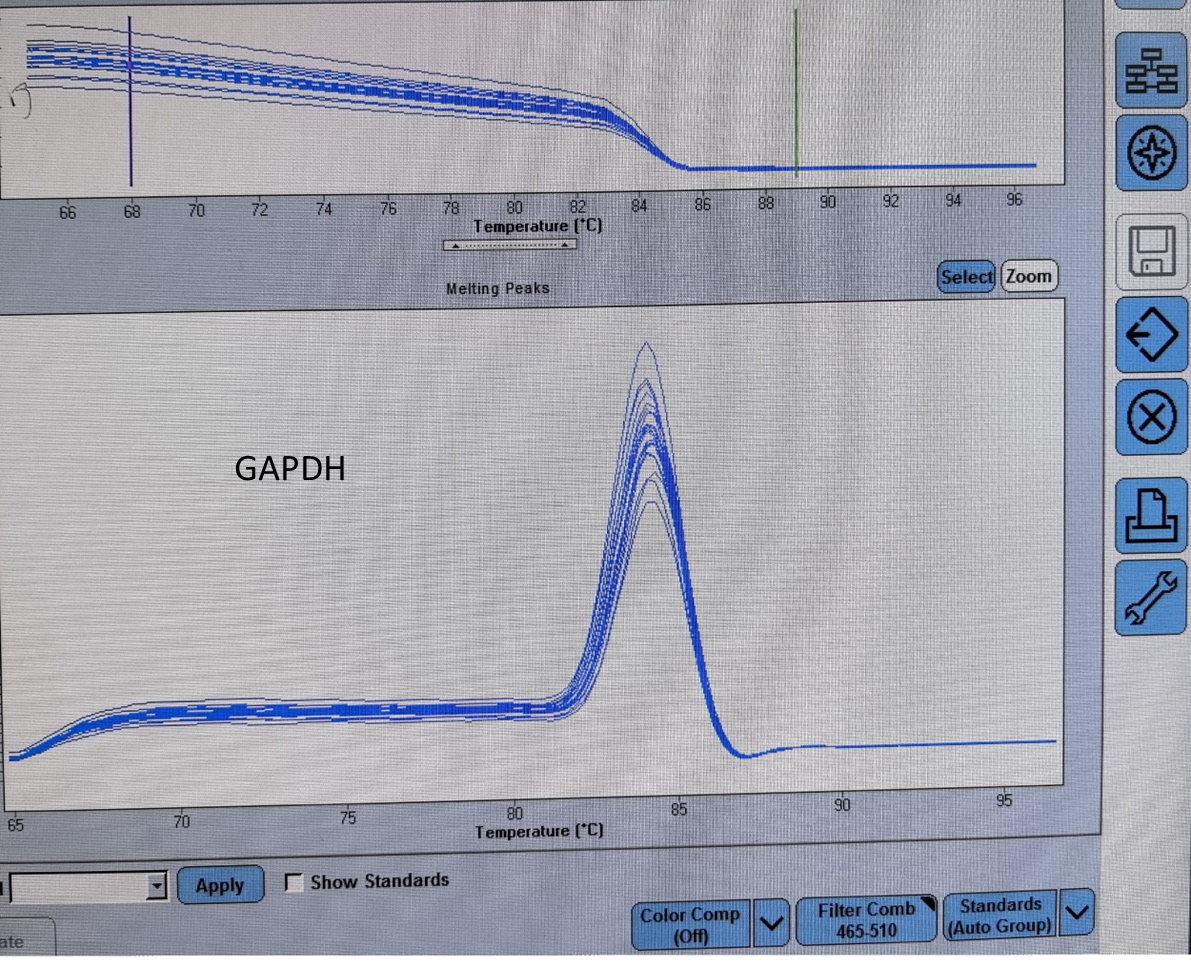


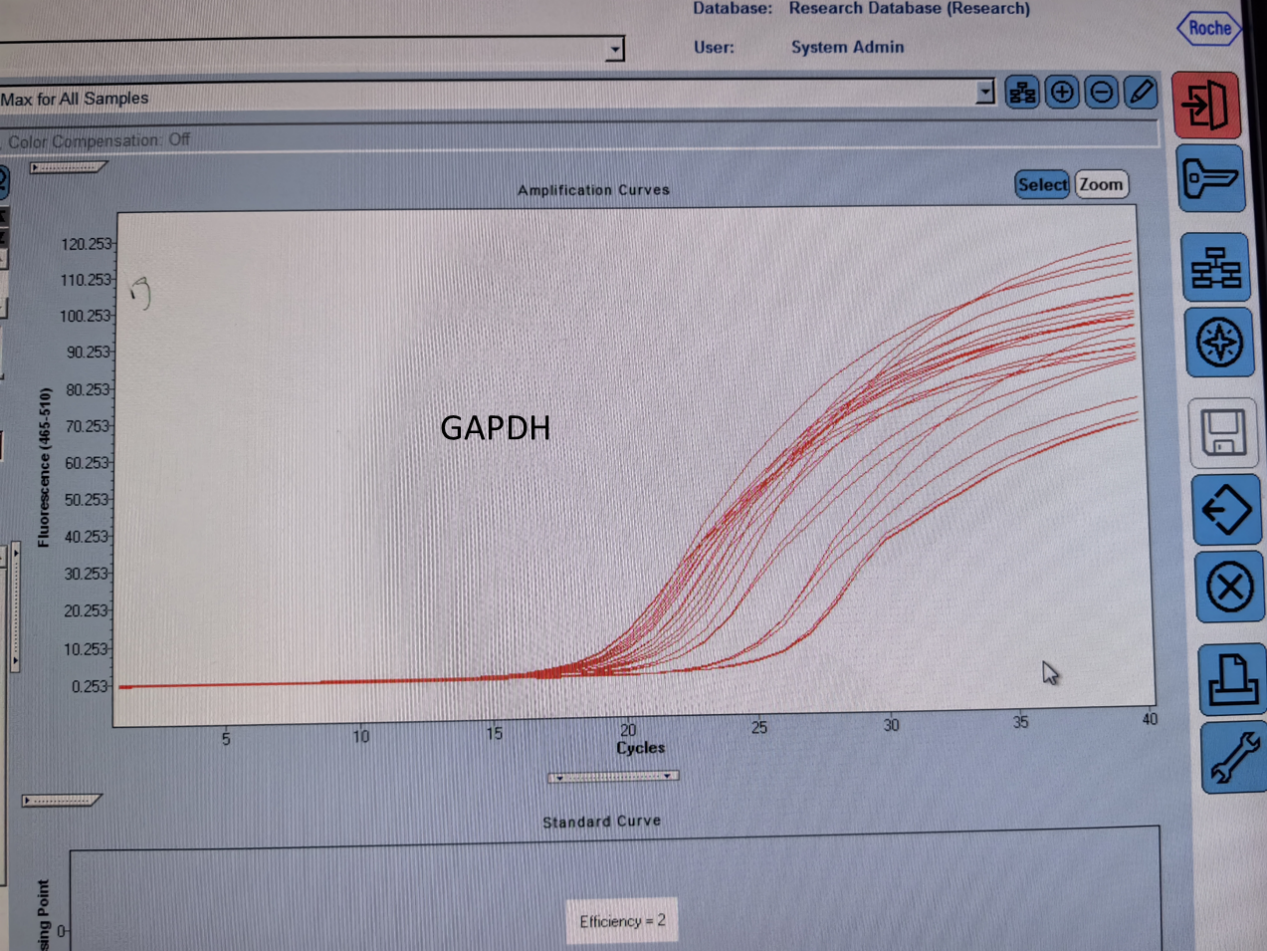


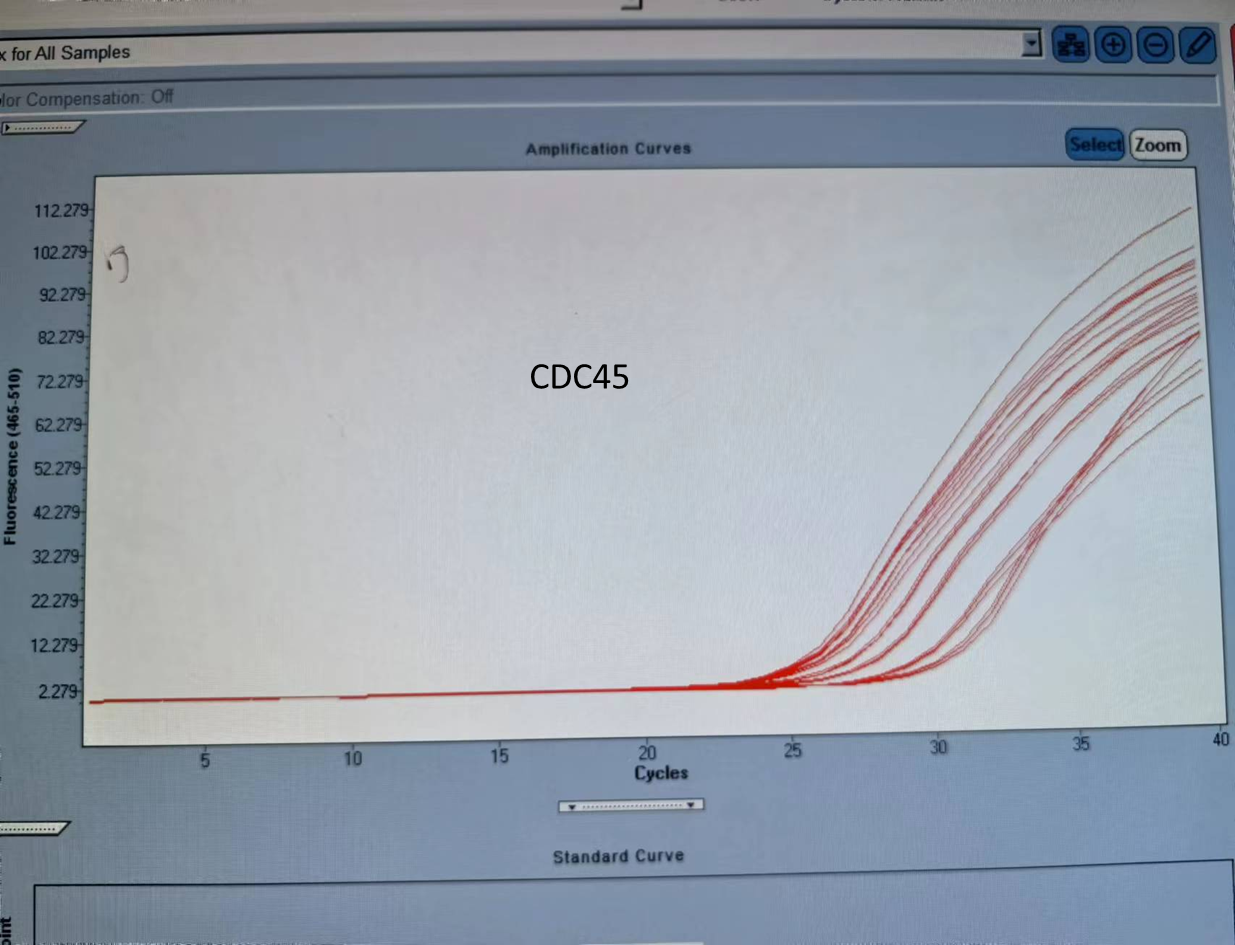

Supplement: Supplemental Information 2 [file peerj-12-17130-s002.zip › RT-qPCR RAWDATA/qPCR amplification and melting curve data for CDC45 and GAPDH.docx]

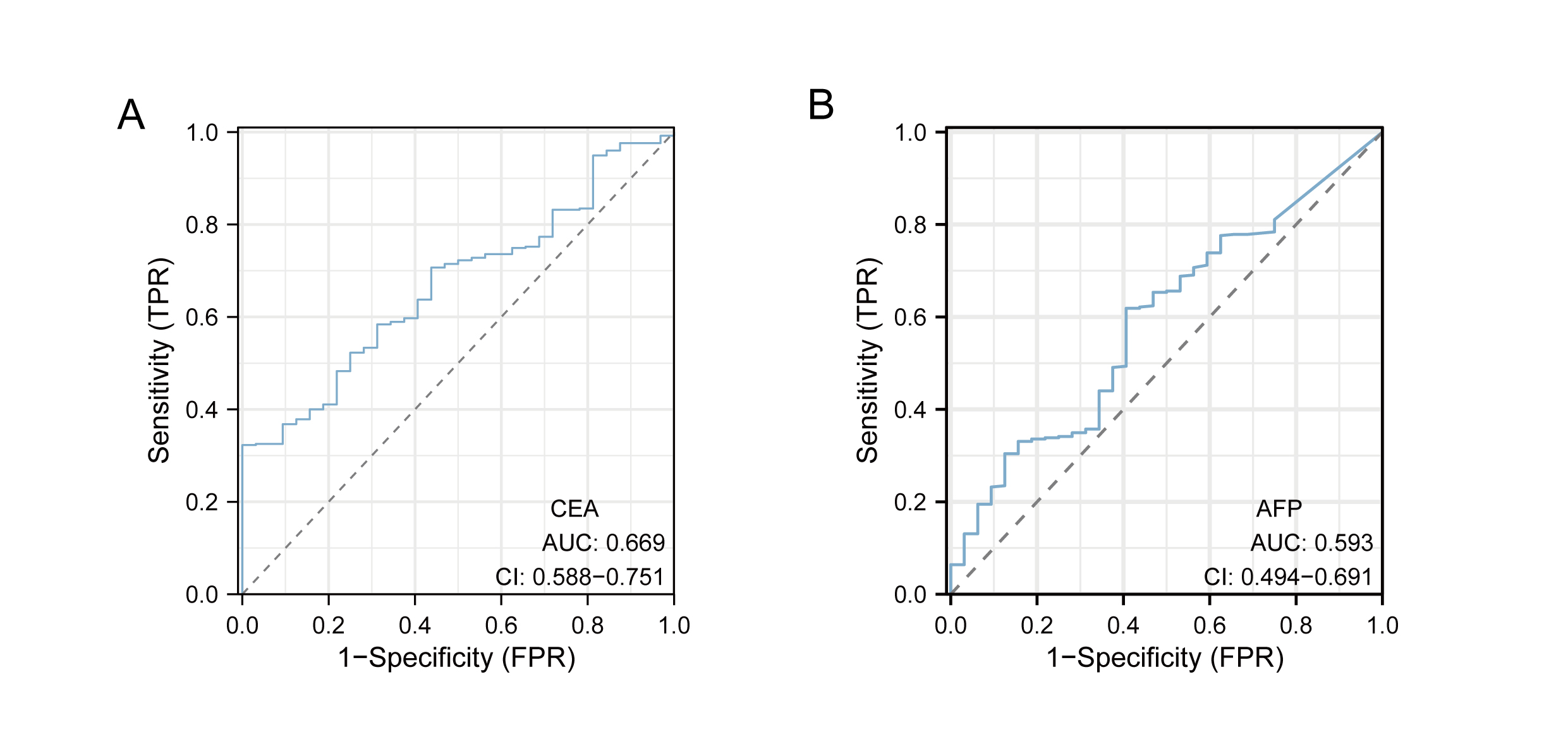

Supplement: Supplemental Information 7 [file peerj-12-17130-s007.jpg]
