# Supplementary material for: Validation of CDC45 as a novel biomarker for diagnosis and prognosis of gastric cancer
Source: PeerJ. 2024 Mar 18;12:e17130. doi: 10.7717/peerj.17130 (PMC10956518; doi:10.7717/peerj.17130)
Supplement: Supplemental Information 8 [file peerj-12-17130-s008.docx]

MIQE Checklist

**1. Experimental Design**

- Provide detailed information.

| **GC** | **GENDER** | **age** | **diagnose** | **Pairs adjacent tissues** |
| --- | --- | --- | --- | --- |
| WA1 | female | 61 | Moderately differentiated adenocarcinoma of gastric horn | AP1 |
| WA2 | male | 53 | Gastric Angle - antrum - superficial poorly differentiated adenocarcinoma of gastric body surface | AP2 |
| WA3 | female | 54 | Gastric adenocarcinoma | AP3 |
| WA4 | female | 75 | Early gastric (0-IIb) moderately differentiated tubular adenocarcinoma, invading the mucous muscle layer | AP4 |
| WA6 | male | 65 | Antrum carcinoma | AP6 |
| WA7 | female | 51 | Antral adenocarcinoma with multiple abdominal metastases (cT4b) | AP7 |
| WA10 | male | 56 | Highly differentiated tubular adenocarcinoma of posterior wall of stomach body | AP10 |
| WA11 | male | 40 | Ulcerated moderately differentiated adenocarcinoma in the lateral curvature of gastric antrum | AP11 |
| WA13 | male | 63 | Early gastric poorly differentiated adenocarcinoma (0-Ⅱc type), partly sig-ring cell carcinoma (20%), | AP13 |
| WA14 | male | 65 | Gastric ulcerative moderately differentiated tubular adenocarcinoma | AP14 |
| WA20 | male | 63 | Early gastric poorly differentiated adenocarcinoma (0-Ⅱc type), | AP20 |
| WA21 | female | 37 | Low adhesion carcinoma, mostly signet-ring cell carcinoma | AP21 |

- Describe the experimental and control groups.

Fluorescence quantitative PCR was used to detect the expression of CDC45 gene in 12 pairs of gastric cancer and adjacent tissues, mainly to analyze the relative expression multiple of CDC45 gene in gastric cancer. See the Methods section“***2.3 RT-PCR analysis***” of the manuscript for details***.***

**2. Sample Handling and RNA Extraction**

- The gastric cancer tissues were preserved in RNAlater solution (Qiagen, USA) at a temperature of -80 °C.

-RNA extraction: The total RNA from these tissues was extracted using the SteadyPure Quick RNA Extraction Kit (Accurate Biology, AG21023, China) following the instructions provided by the manufacturer. See the Methods section“***2.3 RT-PCR analysis***” of the manuscript for details***.***

- Provide the nucleic acid concentration and purity for each sample.

| **NO.** | **A260/A280** | **A260/230** | **(ng/ul)** |
| --- | --- | --- | --- |
| WA1 | 2.02 | 1.84 | 1559.05 |
| AP1 | 1.73 | 0.88 | 330.308 |
| WA2 | 2.04 | 1.99 | 2906.99 |
| AP2 | 1.92 | 1.25 | 910.91 |
| WA3 | 1.87 | 1.71 | 3061.64 |
| AP3 | 1.96 | 1.41 | 782.181 |
| WA4 | 1.81 | 1.49 | 3642.97 |
| AP4 | 1.79 | 1.02 | 1556.41 |
| WA6 | 1.81 | 1.86 | 3536.06 |
| AP6 | 1.79 | 0.88 | 716.709 |
| WA7 | 1.83 | 1.81 | 4110.75 |
| AP7 | 1.65 | 1.43 | 4555.05 |
| WA10 | 1.66 | 1.51 | 494.06 |
| AP10 | 1.92 | 1.47 | 1479.19 |
| WA11 | 1.92 | 1.85 | 1177.06 |
| AP11 | 1.92 | 1.22 | 377.91 |
| WA13 | 1.58 | 1.27 | 2307.21 |
| AP13 | 1.86 | 1.26 | 342.798 |
| WA14 | 1.61 | 1.19 | 3439.62 |
| AP14 | 1.73 | 1.6 | 4016.82 |
| WA20 | 1.81 | 1.69 | 2905.48 |
| AP20 | 1.72 | 1.6 | 4650.48 |
| WA21 | 1.76 | 1.64 | 3952.1 |
| AP21 | 1.76 | 1.1 | 611.595 |

**3. Reverse Transcription**

- The reverse transcription of mRNA was carried out using the GoScript™ Reverse Transcription System (A5001, Promega). See the Methods section“***2.3 RT-PCR analysis***” of the manuscript for details***.***

- Describe the reverse transcription reaction conditions, including reaction volume, temperature, and incubation time.

Reverse transcription uses a 20ul system, and reverse transcription includes 42℃, 15min extension and 70℃, 15min reverse transcriptase inactivation. See the Methods section“***2.3 RT-PCR analysis***” of the manuscript for details***.***

**4. Amplification**

- Specify the amplification reagent used and provide its details.

The mRNA expression levels were measured using the GoTaq® Master Mix (A6001, Promega) following the manufacturer's instructions. See the Methods section“***2.3 RT-PCR analysis***” of the manuscript for details***.***

- Describe the PCR reaction conditions, including reaction components, cycling parameters, and thermal cycling system used.

The measurements were performed on a Roche LightCycler 480 II Real-time fluorescence quantitative PCR machine.

- Provide the details of the CDC45 and GAPDH primer sequences used.Primers were synthesized by Shangya Biotechnology (Fuzhou, China) and GAPDH was used as a reference following the 2−ΔΔCT method. CDC45: forward: 5′-AGAGCATAAAGAACAGTTCCGCTA-3′; reverse, 5′-GACTGGCCTGTGAGTGTCAC-3′.GAPDH Forward primer: 5’-TGCACCACCAACTGCTTAGC-3’; Reverse primer: 5’-GGCATGGACTGTGGTCATGAG-3’. See the Methods section“***2.3 RT-PCR analysis***” of the manuscript for details***.***

**5. Detection and Analysis**

(2−ΔΔCT method). See the Methods section“***2.3 RT-PCR analysis***” of the manuscript for details***.***

**6. Quality Control Measures**

- Describe any quality control measures taken during the experimental process, such as the use of positive and negative controls and experimental replicates.

- Specify how the amplification specificity was ensured (e.g., melting curve analysis).

Using melting curve analysis to control the amplification specificity.
